# Changes in Ginsenoside Composition, Antioxidant Activity and Anti-Inflammatory Activity of Ginseng Berry by Puffing

**DOI:** 10.3390/foods13244151

**Published:** 2024-12-21

**Authors:** You-Jeong Lee, Jae-Sung Shin, Seon-Min Oh, Ji-Eun Bae, Sang-Jin Ye, Hyungjae Lee, Wooki Kim, Moo-Yeol Baik

**Affiliations:** 1 Department of Food Science and Biotechnology, Institute of Life Science and Resources, Kyung Hee University, Yongin 17104, Republic of Korea; dbwjd0115@khu.ac.kr (Y.-J.L.); drumlover@naver.com (J.-S.S.); seonmin@kfri.re.kr (S.-M.O.); wise123@khu.ac.kr (J.-E.B.); lifesci2015@khu.ac.kr (S.-J.Y.); 2 Food Processing Research Group, Korea Food Research Institute, Wanju-gun 55365, Republic of Korea; 3 Department of Food Engineering, Dankook University, Cheonan 31116, Republic of Korea; lee252@dankook.ac.kr; 4 Department of Food and Nutrition, Yonsei University, Seoul 03722, Republic of Korea

**Keywords:** ginseng berry, puffing, ginsenoside, antioxidant activities, anti-inflammatory activities

## Abstract

The effects of puffing on the ginsenoside composition as well as antioxidant and anti-inflammatory activities of ginseng berry were investigated to increase the utilization of ginseng berry. There was no significant difference in extraction yield between the control and puffed samples at all moisture contents and pressure conditions (*p* < 0.05). Major ginsenosides of ginseng berry (especially ginsenoside Re) were degraded through deglycosylation and dehydration by heat and pressure, and new minor ginsenosides (Rg3, F2, Rh2 and Rb2) were produced after puffing. Puffed ginseng berries showed higher total phenolic content (TPC), total flavonoid content (TFC) and Maillard reaction products (MRPs) than those of the control group, and these contents were increased as puffing pressure increased. In addition, higher antioxidant activities were observed in puffed ginseng berries compared to the controls, possibly due to the increase in TPC and MRPs. Antioxidant activity increased with increasing puffing pressure at all moisture contents. Nitric oxide (NO) production showed no significant inhibitory effect between control and puffed ginseng berries (*p* < 0.05). In the case of inflammatory cytokines, IL-6 had an inhibitory effect, but TNF-α had no inhibitory effect. Consequently, puffing showed a positive effect on the composition and the transformation of ginsenosides as well as the antioxidant activity of ginseng berries, suggesting that puffed ginseng berries can be used as a high value-added food material.

## 1. Introduction

The roots of ginseng (*Panax ginseng* C.A. Meyer) have been used as medicinal plants for thousands of years due to their various functions such as anti-diabetic, anti-tumor, antioxidant, and anti-inflammatory activities [1,2,3,4]. Although ginseng stems and leaves affect root development through photosynthesis, the flower rather causes a decrease in root yield through nutrient consumption. Therefore, ginseng berries are either discarded or limitedly utilized [5]. However, recently, a report has shown that ginseng berries contain many polyphenolic substances and have more saponin than the roots, which makes them more functional than ginseng roots [6]. However, ginseng berries are found only in 4-year-old plants and easily and rapidly wither after harvest.

Ginsenoside, a representative active ingredient of the ginseng berry, is a glycoside in which sugar is bound with a basic structure of a derivative of the triterpenoid dammarane. There are more than 150 types of ginsenosides from ginseng roots, leaves, and berries, and their various pharmacological effects such as antioxidant, anti-cancer, and anti-diabetic effects have been recognized [7,8,9,10]. When ginsenosides are subjected to various processing such as heat treatment and fermentation, new ginsenosides, which are not present in the raw materials, are released. These newly released ginsenosides are called minor ginsenosides. Since these minor ginsenosides show better anti-allergic and anti-inflammatory activities, there are many efforts to convert major ginsenosides into minor ginsenosides through various processing methods [11,12].

Puffing is a method of inducing the gelatinization of the starch in a material by suddenly lowering the pressure at high temperatures and initially high pressure, resulting in increased volume as the moisture in the material is removed [13]. During puffing, gelatinization and swelling occur inside the material, resulting in a broken tissue and a porous structure. In addition, a Maillard reaction occurs during puffing and the material’s color becomes dark, and volatile substances are generated, imparting flavor [14]. Furthermore, the shelf life of a material can be improved by reducing the moisture content. Puffing has been reported to increase the extraction yield and antioxidant activity of food materials, such as ginseng, cacao beans, coffee beans, black soy beans, turmeric, wild ginseng and ginger [15,16,17,18,19,20,21,22]. Moreover, puffed turmeric and ginger have been reported to have improved anti-inflammatory activities [21,22]. Puffing also significantly changes the ginsenoside profile in ginseng from major ginsenosides to minor ginsenosides [6,15,18,20] and dramatically converted gingerols to shogaols in ginger [22].

There are studies on the active ingredients and functionality of ginseng berry obtained using various treatments such as steaming or fermentation [23,24], but no study has been performed on puffing ginseng berries. Therefore, in this study, the effects of moisture content and puffing pressure on the changes in ginsenoside, antioxidant activity, and anti-inflammatory activity of ginseng berry were investigated to increase the utilization of ginseng berry in the food and cosmetic industries.

## 2. Materials and Methods

### 2.1. Materials

Ginseng berries (*Panax ginseng* C. A. Meyer) harvested in July 2021 were used (Eumseong, Republic of Korea). Ginseng berries were washed to remove foreign substances and dried at room temperature in a well-ventilated place. The moisture contents of dried ginseng berries were adjusted to 8, 11 and 14% in a dry oven at 40 °C, respectively. Dried ginseng berries were stored at −20 °C for further use.

### 2.2. Chemicals

Gallic acid, catechin, Folin–Ciocalteu’s phenol reagent, ascorbic acid, dimethyl sulfoxide (DMSO), lipopolysaccharide,2,2-diphenyl-1-picrylhydrazyl (DPPH), 2,2′-Azinobis-(3-Ethylbenzthiazolin-6-Sulfonic Acid) (ABTS) and 2,2′-Azobis(2-methylpropionamidine) dihydrochloride (AAPH) were purchased from Sigma-Aldrich Co. (St. Louis, MO, USA). Hydrochloric acid, methyl alcohol, sodium acetate, sodium carbonate and sodium hydroxide were obtained from Daejung Chemicals & Metals Co. (Siheung, Republic of Korea). Dulbecco’s Modified Eagle Medium (DMEM), TNF-α Equine Uncoated ELISA Kit and IL-6 Bovine Uncoated ELISA Kit were purchased from Gibco-Thermo Fisher Scientific Inc. (Waltham, MA, USA). Phosphate buffered saline (PBS) (pH 7.4), 3-(4,5-Dimethylthiazol-2-yl)-2,5-diphenyltetrazolium bromide (MTT) and RAW 264.7 cell line were obtained from Tech & Innovation (Chuncheon, Republic of Korea), Duchefa Biochemie (Haarlem, The Netherlands) and Korea Cell Line Bank (Seoul, Republic of Korea), respectively.

### 2.3. Puffing Process

To minimize the carbonization of ginseng berry due to high temperature, 40 g of ginseng berry and 800 g of rice (1:20 *w*/*w*) were mixed and put into the chamber of a puffing machine and puffed at 588 kPa, 686 kPa and 784 kPa, respectively. Dried, non-puffed ginseng berries were used as a control. Samples were ground using a blender (SFM-353NK, Shinil Industrial Co., Seoul, Republic of Korea) and stored at −20 °C until used.

### 2.4. Extraction

For extraction, 3 g of ginseng berry powder and 60 mL of 70% ethanol (1:20, *w*/*v*) were mixed and extracted with stirring at room temperature for 3 h. Then, the extract was filtered with Whatman # 2 filter paper using a Kimble filtering flask equipped with a funnel. All extracts were sealed and stored at −20 °C. The extraction yield was calculated after drying at 105 °C in an air-drying oven (HB-502M, HanBeak Scientific Co., Bucheon, Republic of Korea) using formula (1):

(1) $$Extraction\;yield\;(\%)=\frac{Weight\;of\;dried\;sample\;(g)}{Initial\;sample\;weight\;(g)}\times \frac{E}{E\prime }\times 100$$

where:

*E*: Total volume of extract (mL);

*E*′: Used volume of extract for drying (mL).

### 2.5. Crude Saponin

The ginseng berry extract was concentrated under reduced pressure at 45 °C, and distilled water was added to make a 5 mL sample. The concentrated sample was moved to a conical tube and then 15 mL of distilled water and 20 mL of diethyl ether were added. This mixture was centrifuged at 3000× *g* for 10 min at room temperature. When the water and ether layers separated, the ether layer was discarded and 20 mL of saturated butanol was added to the remaining water layer. After shaking well, the sample was centrifuged at 3000× *g* for 10 min at room temperature. When the water layer and the butanol layer were divided, the butanol layer was collected separately, saturated butanol was added to the water layer again, and the butanol layer was collected by centrifugation. The collected saturated butanol was concentrated and crude saponin was calculated using formula (2):

(2) $$Crude\;saponin\;(mg/g\;ginseng\;berry)=\frac{Weight\;of\;dried\;sample\;(mg)}{Weight\;of\;total\;sample\;(g)}$$

### 2.6. Ginsenoside Profile

Ginsenoside composition and quantitative analysis were performed using HPLC (Agilent 1260 Infinity II, Santa Clara, CA, USA) equipped with a Kinetex C18 column (4.6 mm × 250 mm, 5 μm) (Phenomenex, Torrance, CA, USA) and a UV detector at 203 nm. Methanol (5 mL) was used to dissolve the concentrated saponin and was filtered using a 0.45 μm Millipore filter, and 5 μL of sample was injected at 45 °C. The flow rate of the mobile phase was 0.6 mL/min. Mobile phases composed of distilled water (Solvent A) and acetonitrile (Solvent B) were added at the following concentrations: Solvents A and B were adjusted at 81:19, 71:29, 60:40, 44:56, 30:70, 10:90 and 81:19 at times of 0~7, 7~14, 14~25, 25~28, 28~30, 30~34 and 34~40 min, respectively.

### 2.7. Maillard Reaction Products

Maillard reaction products (MRPs) were measured using spectroscopic method of Ajandouz et al. [25]. Absorbance was measured at 420 nm using a UV-Vis spectrophotometer (UV-12, LABENTECH Co., Incheon, Republic of Korea). All samples were adjusted to 10 mg/mL and diluted 10 times using an extract solvent (70% ethanol) to maintain an absorbance less than 0.65.

### 2.8. Total Phenolic Content and Total Flavonoid Content

Total phenolic content (TPC) of puffed ginseng berries was determined by Folin and Ciocalteu’s method [26]. Gallic acid was used as a standard material for TPC, and the result was expressed as gallic acid equivalents (GAE)/g dried ginseng berry.

Total flavonoid content (TFC) of puffed ginseng berries was determined by colorimetric method [27]. Catechin was used as a standard material for TFC, and the results were expressed as catechin equivalents (CE)/g dried ginseng berry.

### 2.9. Antioxidant Activity

#### 2.9.1. DPPH Radical Scavenging Activity

DPPH radical scavenging activity of ginseng berry extract was measured by the colorimetric method [28]. Briefly, 50 µL of the extract was added to 2.95 mL of a 0.1 mM DPPH solution, and absorbance at 517 nm was measured after 30 min. Ascorbic acid was used as a control for antioxidant activity, and the result was expressed as vitamin C equivalents (VCE)/g dried ginseng berry.

#### 2.9.2. ABTS Radical Scavenging Activity

ABTS radical scavenging activity of ginseng berry extract was measured by the colorimetric method [29]. Briefly, AAPH, ABTS^2−^, and phosphate buffer saline (PBS) were reacted at 70 °C for 30 min and filtered through a 0.45 µm syringe filter. Then, 20 μL of the extract and 980 μL of the ABTS solution was mixed and reacted at 37 °C for 10 min, after which the absorbance at 734 nm was determined. Ascorbic acid was used as a control for antioxidant activity, and the result was expressed as vitamin C equivalents (VCE)/g dried ginseng berry.

### 2.10. Anti-Inflammatory Activity

#### 2.10.1. Cell Culture

RAW 264.7 cells were seeded at 10^6^ cells/mL in DMEM complete medium including 10% FBS and 1% antibiotic/antimycotic solution. Cell culture was performed in a CO_2_ incubator (BB15, Thermo Scientific, Waltham, MA, USA) with 5% CO_2_ at 37 °C.

#### 2.10.2. Cell Viability Assessment

Cell viability was determined using the MTT method. Briefly, RAW 264.7 cells were seeded in 90 μL DMEM complete medium at density of 3 × 10^4^ cells/m into a 96-well plate, followed by addition of 10 μL ginseng berry extract at designated concentrations. After 24 h of culture, the supernatant was removed, and 100 μL of MTT solution was treated for 3.5 h. Then, cells were washed by addition of excess PBS and centrifugation at 500× *g* and resuspended in 150 μL of DMSO, for which absorbance was quantified at 595 nm by using a Bio-Rad microplate reader (Bio-Rad, Hercules, CA, USA).

#### 2.10.3. Measurement of Nitric Oxide (NO)

The secretion of NO was measured using Griess reagent. Briefly, 500 μL of RAW 264.7 cells at concentration of 1 × 10^6^ cell/mL were seeded in a 24-well plate, and 500 μL of puffed ginseng berry extract was added at designated doses. After 24 h incubation, cells were further stimulated with 10 μL of LPS (lipopolysaccharides) (500 ng/mL) for another 24 h, followed by the harvest of supernatants by centrifugation. Griess reagent was added to the culture supernatants at an equal volume, and absorbance was observed at 540 nm, for which sodium nitrite was used as a standard for NO measurement.

#### 2.10.4. Measurement of TNF-α and IL-6 Production

The cellular secretion of inflammatory cytokines TNF-α and IL-6 was assessed by using ELISA kits. Briefly, captured antibodies in a coating buffer were treated in a 96-well plate and stored overnight at 4 °C. Then, the coating buffer was removed, and the wells were washed 3 times with coating buffer. After incubation in 200 μL assay buffer for 1 h, the plates were washed 3 times with a diluent. Standards at serial dilution or cell culture supernatants were added to the volume of 100 μL. After 2 h incubation, plates were washed 5 times with a diluent, and 100 μL of biotinylated detection antibody and avidin-conjugated horse radish peroxidase were treated for 1 h. After the reaction, plates were washed 7 times with excess diluent, and 100 μL of substrate solution was added per well and kept for 30 min under no light. After adding 50 μL of stop solution, the absorbance was measured at 450 nm.

### 2.11. Statistical Analysis

All experimental results were expressed as the average and standard deviation after 3 repetitions. Multiple comparisons between the means were performed with Tukey’s test at the 5% significance level using the SAS program (version 9.4; SAS Institute, Inc., Cary, NC, USA).

## 3. Results and Discussion

### 3.1. Morphology and Extraction Yield of Control and Puffed Ginseng Berries

**Figure 1:**
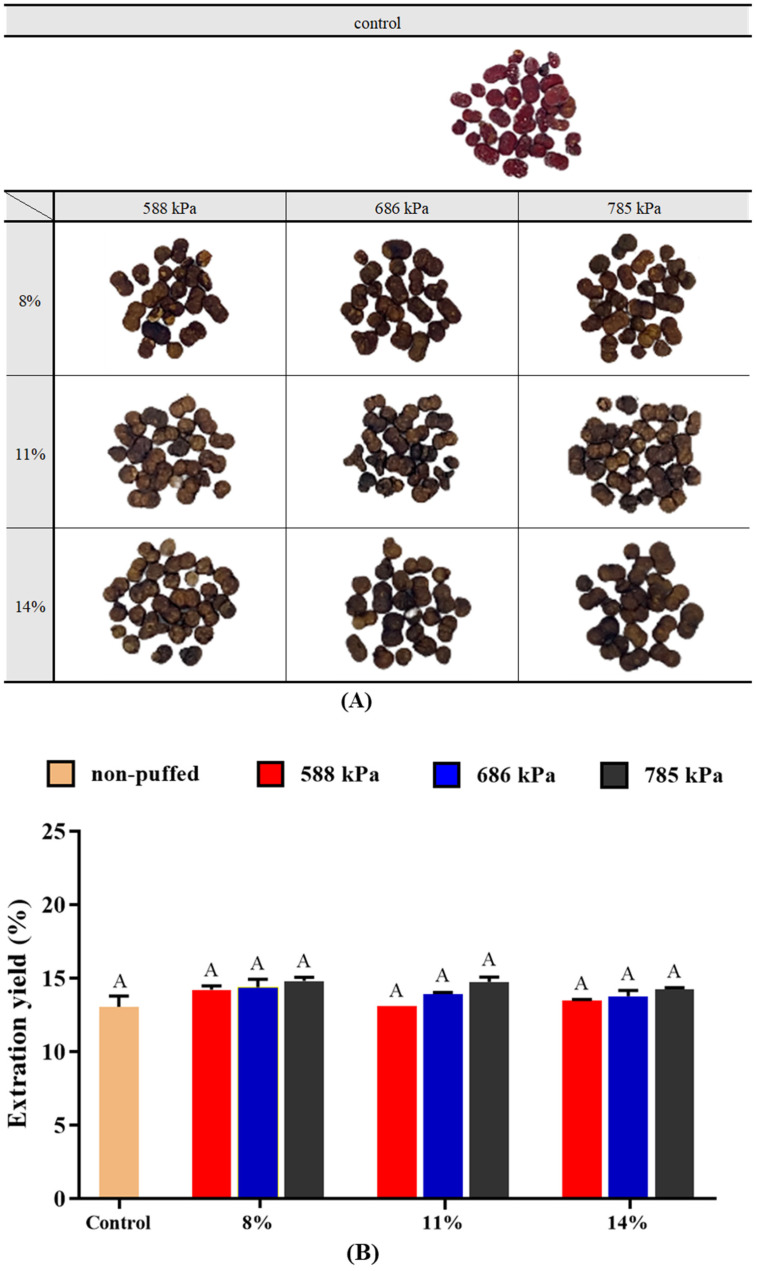
Morphology (**A**) and extraction yield (**B**) of puffed ginseng berries. Different letters above the bars indicate significant difference (*p* < 0.05).

The appearances of puffed ginseng berries obtained using various moisture contents and pressure conditions are shown in Figure 1A. As the puffing pressure increased, non-enzymatic browning increased, and the crushing characteristics of the surface became stronger. Differences in moisture content did not significantly change the appearance of the puffed ginseng berries. In the case of red ginseng root, the volume expands after puffing [15]. However, volume expansion was not observed in ginseng berry. It is assumed that the size of the ginseng berry is too small to undergo volume expansion.

It has been reported that the puffing treatment caused a porous structure to form, resulting in the increase in extraction yield of ginseng, ginger, and black beans [20,21,22]. However, there was no significant difference in extraction yield at all moisture contents and pressure conditions (*p* < 0.05) in ginseng berries (Figure 1B). Due to the relatively small size of the ginseng berry, puffing failed to increase the volume or to produce a porous structure, resulting in no significant difference in extraction yield.

### 3.2. Ginsenoside Profile and Quantification

**Figure 2:**
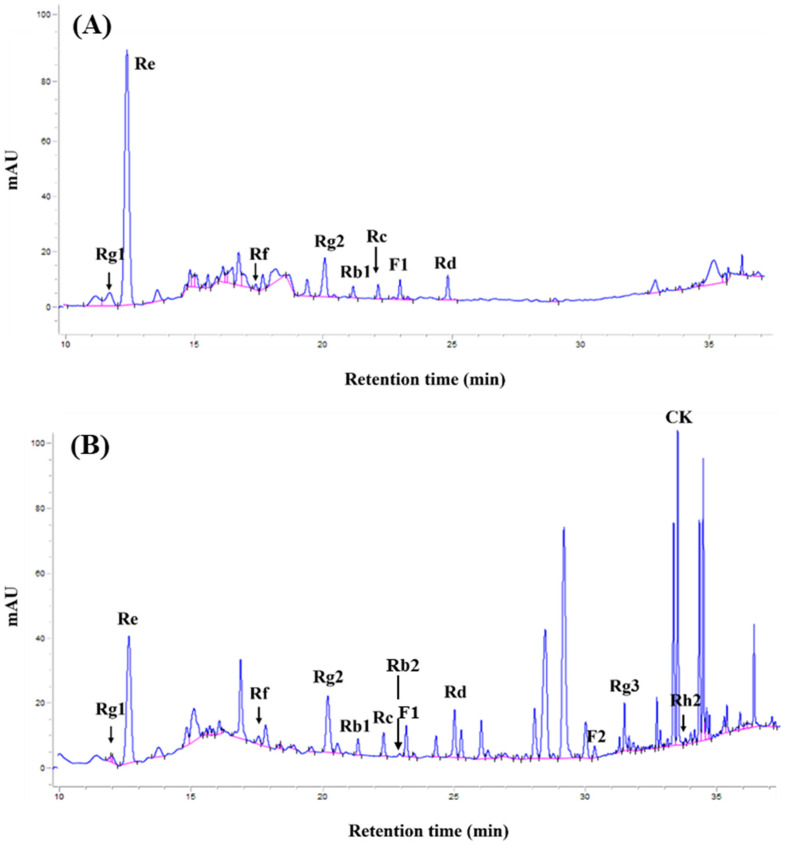
HPLC chromatograms of control (**A**) and puffed ginseng berry (11% moisture content puffed at 785 kPa) (**B**).

The representative ginsenoside Re of ginseng berry showed the highest peak, and minor ginsenoside peaks, such as for Rb2, F2, Rg3 and Rh2, were not detected in the control (Figure 2A). On the other hand, the ginsenoside Re peak was significantly reduced in the puffed ginseng berry, and some minor ginsenoside peaks (Rb2, F2, Rg3 and Rh2) were produced (Figure 2B). These results suggest that high temperature and pressure in the puffing process greatly affected the structural transformation or conversion of ginsenosides. High temperature has been known to promote dehydration and deglycosylation of major ginsenosides and to produce minor ginsenosides [15]. The representative ginsenoside Re of ginseng berry has been reported to be converted into Rg1, F1, Rf and Rg2 through deglycosylation and dehydration [30,31]. It has been reported that ginsenoside Rb1 is decomposed into Rd, F2 and CK, and ginsenosides Rc, Rb2 and Rd are decomposed into Rg3, Rg5 and Rk1 through dehydration and deglycosylation during heat treatment [11,32].

The quantitative changes in ginsenosides are shown in Table 1. The sums of measured ginsenoside contents were increased at 8% and 14% moisture contents compared to the control. Meanwhile, the sum of ginsenoside content was decreased at a 11% moisture content. On the other hand, the 11% and 14% moisture contents revealed relatively higher crude saponin contents, and the 8% moisture content sample showed relatively lower crude saponin contents than that of the control. This discrepancy between the sum of ginsenoside content and crude saponin content may possibly be due to the use of a limited number of standard ginsenoside samples. In this study, only 13 standard ginsenosides were quantitatively analyzed, and other ginsenosides without standards were not included in the analysis results. Therefore, it is not necessary to discuss the sum of ginsenoside contents in this case. However, it can be suggested that the ginsenoside composition can be controlled by changing the moisture content and puffing pressure.

Among the major ginsenosides, the sum of protopanaxadiol (Rb1, Rb2, Rc, Rd) types increased by 160–340%, and the sum of protopanaxatriol (Re, Rg1, Rf) types decreased by 44–81%. Also, minor ginsenosides (F2, Rg3, CK and Rh2) were not detected in the control, but detected after puffing. The increased content of the PPD type is considered to have resulted in the production of ginsenosides Rb1, Rb2, Rc and Rd due to the demalonization of acid malonyl ginsenosides Rb1, Rb2, Rc and Rd, in which the carboxyl group of malonic acid is ester-linked upon heat treatment [33,34], and minor ginsenosides are generated due to hydrolysis and dehydration at the carbon 20 of major ginsenosides (Rg1, Re, Rf, Rb1, Rb2, Rc and Rd) [35]. Minor ginsenosides are known to be easier to absorb in the human body than major ginsenosides and have potent pharmacological effects such as anti-cancer and antioxidant activities [8,12]. It has been reported that ginsenoside Rg3 has anti-cancer effects and CK has anti-cancer and anti-inflammatory effects [8,36]. The highest content of ginsenoside Rg3 and compound K was observed in the sample with an 8% water content and puffed at 686 kPa. However, there was no correlation between moisture content and puffing pressure.

### 3.3. Maillard Reaction Products (MRPs)

**Figure 3:**
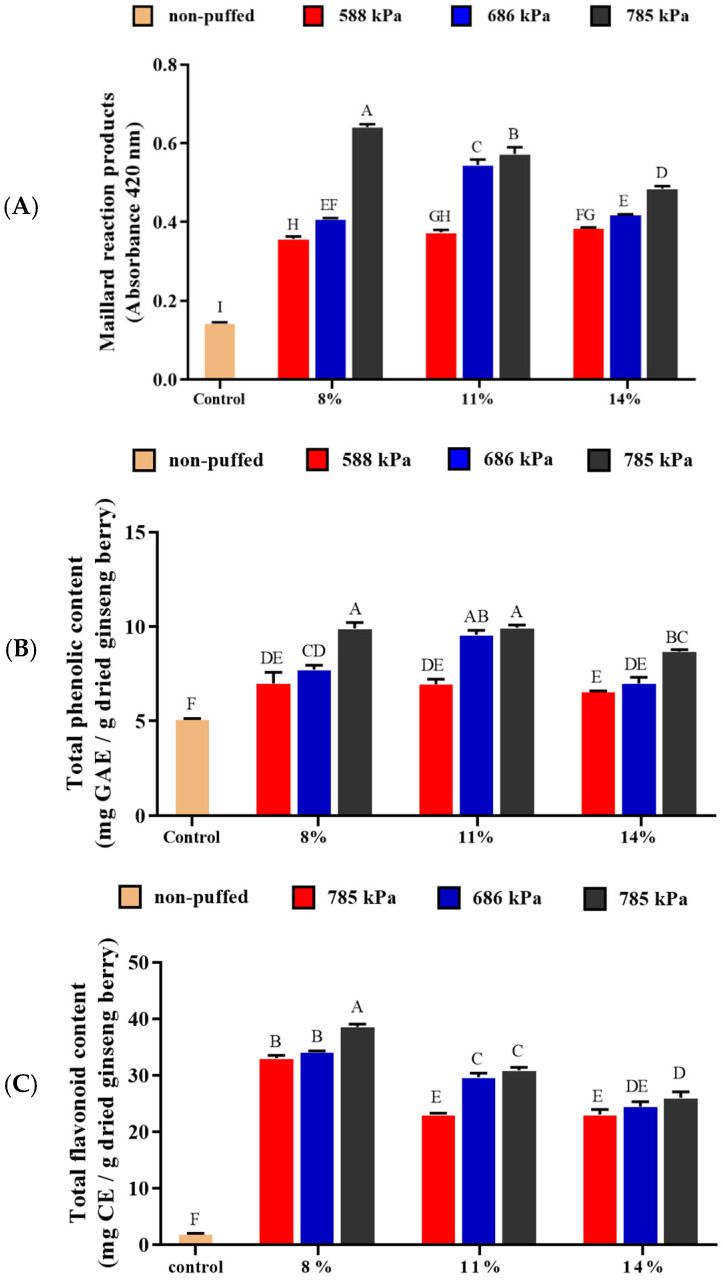
Changes in Maillard reaction products (**A**), total phenolic contents (**B**) and total flavonoid contents (**C**) of puffed ginseng berries. Different letters above the bars indicate significant difference (*p* < 0.05).

The amount of MRPs increased as the puffing pressure increased in all moisture conditions (Figure 3A). The highest value was 0.641 ± 0.007 at a moisture content of 8% and a pressure of 785 kPa, which was about 5 times higher than that of the control (0.143 ± 0.002). It has been reported that MRPs are formed when ginsenoside Re is heat-treated with alanine, lysine and leucine at a temperature higher than 100 °C [37,38,39]. It was also observed that the sugars that separate from Rb1 and Rb2 were converted into Rg3 and Rg5 by deglycosylation and dehydration during the steam process and react with glycine to form MRPs [40,41]. Therefore, the increase in MRPs in this study suggests that the Maillard reaction between the sugars and the amino acids progressed actively as the sugars of ginsenoside were decomposed by a high temperature and pressure.

### 3.4. Total Phenolic Content (TPC) and Total Flavonoid Content (TFC)

Total phenolic content (TPC) increased with increasing pressure at all moisture contents (Figure 3B). TPC was the highest in the ginseng berry with 11% moisture content puffed at 785 kPa (9.94 ± 0.14 mg GAE/g dried ginseng berry), which was 195% higher than that of control (5.09 ± 0.04 mg GAE/g dried ginseng berry). Phenolic substances found in plants are known to have excellent antioxidant activity, and the TPC has been reported to be increased when puffing was applied to cultivated wild ginseng [17], turmeric [16], *Platycodon grandiflorum* roots [42], American and Canadian ginsengs [30] and coffee beans [19]. It was also observed that the TPC of Korean ginseng increased when treated with high temperature and high pressure at 110 °C and 981 kPa, respectively [43]. When mango seeds were heated, their TPC also increased [44]. The increases in TPC may be explained by the weakening of the molecular binding as a result of heat and pressure treatment and the elution of those materials into a water-soluble state as well as the increase in certain substances such as maltol, a Maillard reaction product [30,45]. The TPC has been reported to increase with increasing temperature and heat treatment time [30,45]. In addition, since polyphenol levels have been reported to increase in the later stages of the Maillard reaction, it may be due to an increase in MRPs [45].

The total flavonoid content (TFC) also increased with increasing pressure at all moisture contents (Figure 3C). The TFC was the highest in the ginseng berry with 8% moisture content puffed at 785 kPa (38.57 ± 0.51 mg CE/g dried ginseng berry), which was 3589% higher than that of the control (1.09 ± 0.07 mg CE/g dried ginseng berry). Flavonoids are one of the secondary metabolites of plants. When heat is applied to plants, the flavonoid-containing macromolecules are broken down into smaller molecules, increasing the total amount of flavonoids [46]. Moreover, it was reported that high-pressure homogenization at 250 MPa for 10 min increased the content of individual flavonoids in apple, orange and grape juice due to the disruption of cellular structures [47]. Therefore, the increase in the TFC in this study could be due to the destruction of the cell structure and degradation of flavonoid-containing polymers as a result of heat and pressure during puffing.

### 3.5. Antioxidant Activities

**Figure 4:**
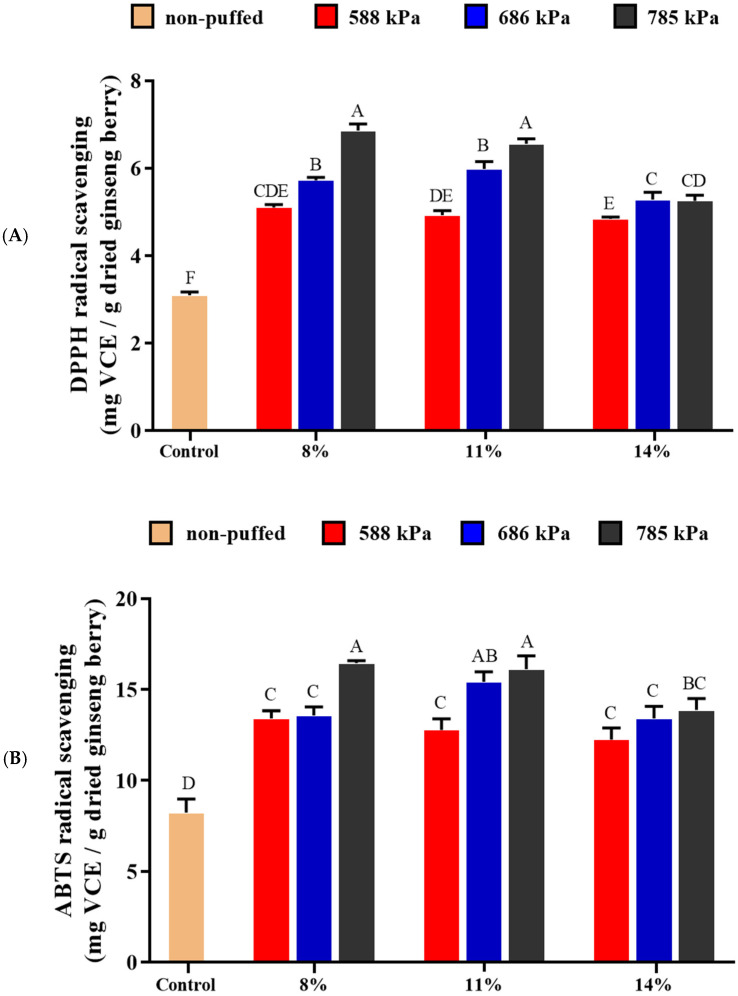
Antioxidant activities of puffed ginseng berries: (**A**) DPPH radical scavenging activity, (**B**) ABTS radical scavenging activity. Different letters above the bars indicate significant difference (*p* < 0.05).

Antioxidant activities of puffed ginseng berry were determined using DPPH and ABTS radical scavenging activities (Figure 4). The DPPH radical is widely used to evaluate antioxidant activity in a short time, and puffed ginseng berries showed higher DPPH radical scavenging activities compared to the control (Figure 4A). As the pressure increased, the radical scavenging activity was further enhanced. The highest value was 6.86 ± 0.15 mg VCE/g of dried ginseng berry with 8% moisture content puffed at 785 kPa, which was 222% higher than that of the control (3.09 ± 0.08 mg VCE/g dried ginseng berry). Similar to the DPPH radical scavenging activity, the ABTS scavenging activity also increased as the pressure increased (Figure 4B). The highest value was 16.45 ± 0.14 mg VCE/g of dried ginseng berry with 8% moisture content puffed at 785 kPa, which was 200% higher than that of the control (8.23 ± 0.76 mg VCE/g dried ginseng berry). Polyphenols and flavonoids are known to have DPPH and ABTS radical scavenging activities [48,49,50]. Therefore, these results are considered to be closely associated with increased TPC and TFC in puffed ginseng berries. In addition, MRPs have been reported to also have DPPH, ABTS, and FRAP radical scavenging effects [51]. The free radical-scavenging activity of the ginsenoside Re–lysine mixture was increased by MRPs generated during heat treatment [39]. Therefore, it is regarded that the increase in MRPs in puffed ginseng berries also affected the improvement in free radical scavenging activities.

### 3.6. Anti-Inflammatory Activity

#### 3.6.1. Cell Viability

**Figure 5:**
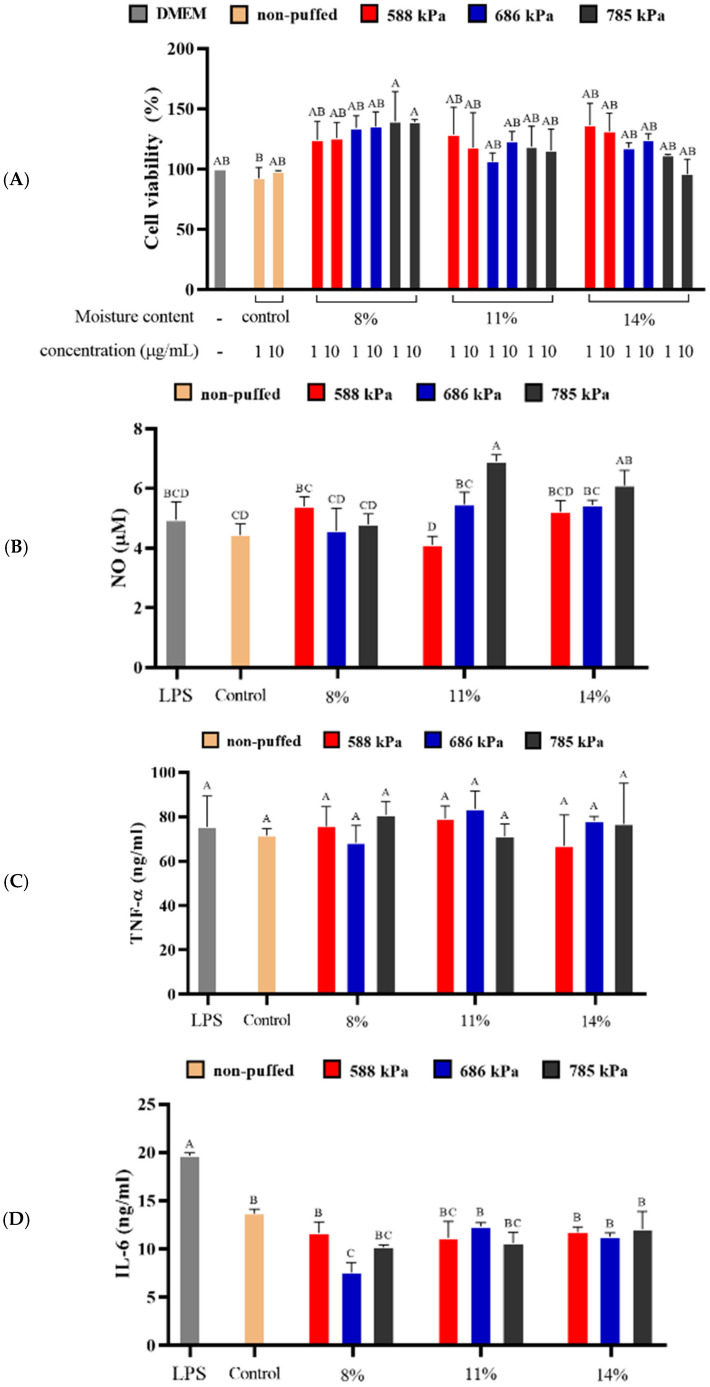
Anti-inflammatory activities of puffed ginseng berries: (**A**) cytotoxicity, (**B**) NO production, (**C**) TNF-α production, (**D**) IL-6 production. Extracts were treated at 10 µg/mL. Different letters above the bars indicate significant difference (*p* < 0.05).

The viability of murine RAW 264.7 cell-line following treatment of puffed ginseng berry extracts was evaluated by MTT assay, in which mitochondrial reductase of living cells produces formazane crystals for quantification by optical observance [52]. As shown in Figure 5A, cell viabilities of 92–130% were observed after treatment of puffed ginseng berry extracts, without any statistical significance, as compared to DMEM-treated control cells (*p* > 0.05), indicating that ginseng berry extracts, regardless of puffing process, are non-toxic to cells. Therefore, the extracts at a concentration of 10 µg/mL were used in the following anti-inflammatory activity assessments.

#### 3.6.2. Production of Nitric Oxide (NO)

The effect of puffed ginseng berry extracts on the nitric oxide (NO) production in LPS-activated RAW264.7 cells is shown in Figure 5B. The highest NO production was observed in the 11% moisture content ginseng berry puffed at 785 kPa (6.90 ± 0.22 µM), and there were no significant differences between cells treated with LPS only and those treated with the other extracts (*p* > 0.05). This result indicates that there was no beneficial role of ginseng berry extract treatment for the production of the inflammatory mediator NO in macrophages. NO is well known for its involvement in acute inflammatory responses in host defense and immunity. However, chronic inflammation results in repetitive NO production and accumulation, which further stimulates oxidative stress and can promote chronic diseases, including tumors, by causing cellular damage [53]. Therefore, its inhibition has been widely studied to prevent chronic inflammation, and 70% ethanol extract of ginseng berry exhibited anti-inflammatory effect [54]. The discrepancy of the current observation might come from the difference in dose of extracts. The aforementioned Kim and Ko study [54] used extracts at concentration of 0.25–1 mg/mL, which is a much higher dose that that of the current work at 10 µg/mL.

#### 3.6.3. Production of Inflammatory Cytokine (TNF-α and IL-6)

The effect of each puffed ginseng berry extract on the production of inflammatory cytokines, i.e., TNF-α and IL-6, in LPS-induced RAW264.7 cells is shown in Figure 5C,D. There was no significant difference in TNF-α production in all extract-treated groups as compared to cells treated with LPS only (*p* > 0.05). On the other hand, IL-6 production was decreased in cells treated with non-puffed control extract as compared to cells treated with LPS only, indicating the anti-inflammatory properties of ginseng berry. Furthermore, puffed ginseng berry at 8% moisture content and 686 kPa showed a further decrement in IL-6 production by inflammatory cells at 7.53 ± 1.04 ng/mL compared to cells treated with LPS only (19.65 ± 0.31 ng/mL).

Like NO, inflammatory cytokines result in chronic inflammatory cues, causing diseases such as cancer and rheumatoid arthritis [55]. In this regard, it has been reported that ginseng berry extract suppresses TNF-α and IL-6 in LPS-induced RAW264.7 cells and exhibits anti-inflammatory activity [56]. However, the current study shows that there are no TNF-α inhibitory effects of ginseng berry extracts, regardless of puffing process, for which the low dose might be relevant. In the case of IL-6, however, it was shown that ginseng berry extracts, regardless of puffing process, have an inhibitory effect even at a lower dose. In addition, a specific puffing condition might be more beneficial for improving its anti-inflammatory properties. Overall, the anti-inflammatory activity of puffed ginseng berry was only observed in IL-6 in this case, and the lack of an inhibitory effect on NO and TNF-α production might be due to the use of relatively low concentrations.

## 4. Conclusions

The effect of puffing on the ginsenoside composition, antioxidant activity and anti-inflammatory activity of ginseng berry was investigated. Upon puffing of ginseng berry, the extraction yield was not significantly changed, and significant browning occurred after puffing, resulting in an increase in MRPs in puffed ginseng berry. The major ginsenosides were decomposed into minor ginsenosides, suggesting that the composition of ginsenosides in ginseng berry can be controlled by adjusting the water content and puffing pressure. Puffing also increased both the TPC and TFC of ginseng berry, and these contents are increased with increasing pressure at all moisture contents. Similar to the TPC and TFC, antioxidant activities, determined by DPPH and ABTS radical scavenging activities, also increased after puffing, and they were improved with increasing pressure at all moisture contents. In the case of anti-inflammatory activity, the inhibitory effects of NO and TNF-α were not significant, possibly due to relatively low concentration of the used ginseng berry extract. On the other hand, an inhibitory effect on IL-6 was observed even at low concentrations, but no significant difference was confirmed with puffing, suggesting that puffing did not affect the anti-inflammatory activity of ginseng berry. Overall, puffing showed a positive effect on the composition and the transformation of ginsenosides, as well as the antioxidant activity of ginseng berry. Therefore, ginseng berries that have undergone puffing can be used as a high value-added material.

## Figures and Tables

**Table 1:** Ginsenoside profiles of puffed ginseng berries.

Ginsenoside (mg/g Dried Ginseng Berry)															Crude Saponin
	Rg1	Re	Rf	Rg2	Rb1	Rc	Rb2	F1	Rd	F2	Rg3	CK	Rh2	SUM	(mg/g D.B)
**Control**	1.92 ± 0.1 ^B^*	43.53 ± 0.7 ^A^	0.63 ± 0.1 ^G^	5.33 ± 0.1 ^F^	1.27 ± 0.2 ^E^	1.46 ± 0.0 ^F^	ND **	1.29 ± 0.2 ^G^	2.11 ± 0.2 ^H^	ND	ND	0.11 ± 0.0 ^I^	ND	57.01 ± 1.2 ^E^	148.47 ± 13.4 ^CDE^
**8%-588 kPa**	2.66 ± 0.3 ^A^	33.59 ± 0.3 ^C^	0.92 ± 0.0 ^E^	8.50 ± 0.5 ^CD^	3.36 ± 0.1 ^A^	4.79 ± 0.2 ^A^	0.71 ± 0.1 ^A^	3.17 ± 0.1 ^AB^	6.62 ± 0.1 ^B^	0.81 ± 0.0 ^D^	1.53 ± 0.0 ^E^	3.67 ± 0.0 ^E^	0.08 ± 0.0 ^F^	70.41 ± 1.2 ^B^	118.51 ± 1.7 ^DE^
**8%-686 kPa**	1.22 ± 0.1 ^CD^	28.82 ± 0.4 ^E^	1.35 ± 0.0 ^A^	12.75 ± 0.3 ^A^	3.29 ± 0.1 ^AB^	4.77 ± 0.2 ^A^	0.58 ± 0.0 ^AB^	3.41 ± 0.2 ^A^	7.85 ± 0.1 ^A^	1.17 ± 0.0 ^A^	4.12 ± 0.1 ^A^	10.89 ± 0.1 ^A^	0.18 ± 0.0 ^CD^	80.39 ± 0.8 ^A^	109.26 ± 10.1 ^E^
**8%-785 kPa**	1.67 ± 0.1 ^BC^	25.66 ± 0.3 ^G^	1.22 ± 0.1 ^B^	9.55 ± 0.1 ^B^	2.65 ± 0.0 ^CD^	4.06 ± 0.2 ^B^	0.62 ± 0.0 ^AB^	2.79 ± 0.0 ^CD^	6.69 ± 0.1 ^B^	0.86 ± 0.0 ^C^	2.59 ± 0.0 ^B^	6.31 ± 0.0 ^B^	0.15 ± 0.0 ^E^	64.82 ± 0.4 ^C^	135.78 ± 14.1 ^DE^
**11%-588 kPa**	1.25 ± 0.0 ^CD^	26.92 ± 0.2 ^F^	0.78 ± 0.0 ^F^	5.17 ± 0.4 ^F^	2.30 ± 0.1 ^D^	3.25 ± 0.2 ^D^	0.50 ± 0.0 ^BC^	2.19 ± 0.1 ^F^	4.50 ± 0.2 ^E^	0.64 ± 0.0 ^F^	0.56 ± 0.0 ^H^	1.48 ± 0.0 ^H^	0.10 ± 0.0 ^F^	49.64 ± 1.1 ^F^	148.35 ± 4.4 ^CDE^
**11%-686 kPa**	0.94 ± 0.0 ^D^	18.48 ± 0.2 ^H^	0.73 ± 0.0 ^F^	5.64 ± 0.1 ^F^	1.57 ± 0.0 ^E^	2.34 ± 0.1 ^E^	0.34 ± 0.1 ^C^	1.57 ± 0.1 ^G^	3.48 ± 0.0 ^G^	0.55 ± 0.0 ^G^	1.34 ± 0.0 ^F^	3.45 ± 0.0 ^F^	0.16 ± 0.0 ^DE^	40.58 ± 0.2 ^G^	228.01 ± 19.2 ^A^
**11%-785 kPa**	1.03 ± 0.2 ^D^	19.64 ± 0.2 ^H^	0.91 ± 0.0 ^E^	7.66 ± 0.0 ^E^	1.57 ± 0.1 ^E^	2.36 ± 0.2 ^E^	0.35 ± 0.0 ^C^	1.59 ± 0.1 ^G^	3.92 ± 0.0 ^F^	0.69 ± 0.0 ^E^	2.03 ± 0.0 ^D^	5.44 ± 0.1 ^C^	0.23 ± 0.0 ^B^	47.42 ± 0.2 ^F^	213.52 ± 10.6 ^AB^
**14%-588 kPa**	1.62 ± 0.2 ^BC^	35.92 ± 0.6 ^B^	1.13 ± 0.0 ^CD^	7.73 ± 0.4 ^E^	2.97 ± 0.2 ^BC^	4.49 ± 0.1 ^A^	0.68 ± 0.1 ^A^	3.03 ± 0.1 ^BC^	6.72 ± 0.1 ^B^	0.90 ± 0.0 ^B^	1.14 ± 0.1 ^G^	2.74 ± 0.0 ^G^	0.16 ± 0.0 ^DE^	68.71 ± 1.0 ^B^	158.21 ± 0.2 ^CD^
**14%-686 kPa**	2.96 ± 0.2 ^A^	30.28 ± 0.6 ^D^	1.08 ± 0.0 ^D^	7.84 ± 0.2 ^DE^	2.47 ± 0.1 ^D^	3.88 ± 0.1 ^BC^	0.62 ± 0.0 ^AB^	2.62 ± 0.1 ^DE^	5.89 ± 0.1 ^C^	0.84 ± 0.0 ^CD^	1.50 ± 0.0 ^E^	3.64 ± 0.1 ^E^	0.21 ± 0.0 ^BC^	62.85 ± 1.3 ^CD^	238.60 ± 11.6 ^A^
**14%-785 kPa**	1.67 ± 0.2 ^BC^	26.49 ± 0.2 ^FG^	1.19 ± 0.0 ^BC^	9.09 ± 0.2 ^BC^	2.30 ± 0.2 ^D^	3.55 ± 0.2 ^CD^	0.64 ± 0.1 ^AB^	2.44 ± 0.1 ^EF^	5.53 ± 0.0 ^D^	0.88 ± 0.0 ^BC^	2.17 ± 0.1 ^C^	5.17 ± 0.0 ^D^	0.30 ± 0.0 ^A^	61.42 ± 0.3 ^D^	182.43 ± 3.3 ^BC^

* Values with the same letter in the same column are not significantly different (*p* < 0.05). ** ND means not detected. Different letters above the bars indicate significant difference (*p* < 0.05).

## Data Availability

The original contributions presented in the study are included in the article, further inquiries can be directed to the corresponding author.
